# Molecular typing of *Treponema pallidum* isolates from Buenos Aires, Argentina: Frequent Nichols-like isolates and low levels of macrolide resistance

**DOI:** 10.1371/journal.pone.0172905

**Published:** 2017-02-24

**Authors:** Lucía Gallo Vaulet, Linda Grillová, Lenka Mikalová, Ricardo Casco, Marcelo Rodríguez Fermepin, María A. Pando, David Šmajs

**Affiliations:** 1 Universidad de Buenos Aires, Facultad de Farmacia y Bioquímica, Departamento de Bioquímica Clínica, Cátedra de Microbiología Clínica, Buenos Aires, Argentina; 2 Universidad de Buenos Aires, Instituto de Fisiopatología y Bioquímica Clínica (INFIBIOC), Buenos Aires, Argentina; 3 Department of Biology, Faculty of Medicine, Masaryk University, Brno, Czech Republic; 4 Universidad de Buenos Aires, Hospital de Clínicas “José de San Martín”, Programa de Enfermedades de Transmisión Sexual (PETS), Buenos Aires, Argentina; 5 Instituto de Investigaciones Biomédicas en Retrovirus y SIDA (INBIRS), Universidad de Buenos Aires-CONICET, Buenos Aires, Argentina; University of Kentucky College of Medicine, UNITED STATES

## Abstract

A total of 54 clinical samples, including genital lesion swabs, whole blood and cerebrospinal fluid from patients diagnosed with syphilis were collected in 2006 and in 2013 in Buenos Aires, Argentina. Treponemal DNA was detected in 43 of the analyzed samples (79.6%) and further analyzed using Sequencing-based molecular typing (SBMT) and Enhanced CDC-typing (ECDCT). By SBMT, 10 different *Treponema pallidum* subsp. *pallidum* (TPA) genotypes were found, of which six were related to the TPA SS14 strain, and four to the TPA Nichols strain. The 23S rRNA gene was amplified in samples isolated from 42 patients, and in six of them (14.3%), either the A2058G (four patients, 9.5%) or the A2059G (two patients, 4.8%) mutations were found. In addition to Taiwan, Madagascar and Peru, Argentina is another country where the prevalence of Nichols-like isolates (26.8%) is greater than 10%.

## Introduction

Syphilis is a sexually transmitted disease caused by *Treponema pallidum* subsp. *pallidum* (TPA). Even though syphilis is a curable disease, the incidence remains at high levels around the world. In 2012 the World Health Organization estimated a global incidence of 5.6 million new syphilis cases in women and men aged 15–49 years worldwide and nearly one million new cases in the Region of the Americas [1]. In Argentina, several studies have identified populations vulnerable to syphilis. In men who have sex with men (MSM) from Buenos Aires, syphilis prevalence ranged from 16.9% to 20.5% [2, 3]. Similarly, high syphilis prevalence has been found among female sex workers (22.4%) [4], and an even higher prevalence among male to female transgender from Buenos Aires (50%) [5]. Low syphilis prevalence was detected among blood donors ranging from 5.3% to7.7% [6, 7].

The genetic characteristics of TPA have been of increasing interest over the past years as an important tool to better understand the epidemiology and network transmission of syphilis infections [8, 9]. Moreover, molecular characterization could be an important tool in the future given the relatively recent emergence of macrolide resistant strains [10].

The Centers for Disease Control and Prevention (CDC, USA) recommend a typing scheme based on the determination of the number of 60 bp-long repeats in the *arp* gene and on the restriction fragment length polymorphism analyses of *tpr*E, *tpr*G, and *tpr*J genes [11]. Recently, sequencing of a portion of the TP0548 gene was added to CDC typing (ECDCT), which increased the discriminatory power of this typing scheme. ECDCT currently includes analysis of *arp* gene, *tprE*, *G* and *J* genes, and TP0548 gene [12]. An alternative molecular typing scheme for TPA was introduced by Flasarová *et al*. in 2006 [13], which was based on sequencing of selected sections of genes TP0136 and TP0548 and selected coordinates in the 23S rRNA gene to reveal macrolide resistance markers [13–15]. While ECDCT is used worldwide and many typing studies have been published using this scheme, SBMT has until now only been used in the Czech Republic and Belgium. One of the benefits of SBMT is that it enables the creation of phylogenetic or network trees to trace syphilis infection.

The aim of this study was to characterize TPA isolates collected in Buenos Aires, Argentina with both Enhanced CDC-typing (ECDCT) and Sequencing-based typing (SBMT).

## Methods

### Clinical samples

Clinical samples were collected from patients with syphilis diagnosed at the Hospital de Clínicas “José de San Martín”, Universidad de Buenos Aires, Argentina. Between March and October 2013, 40 samples were collected including 33 genital lesion swabs, four cerebrospinal fluid samples (CSF), and three whole blood samples. In addition, 14 genital lesion swabs were collected between August and October 2006 and included in this study. Patients suspected of having syphilis based on clinical findings were diagnosed with laboratory tests that included Dark field examination of lesion material and serological tests. Serology included the Venereal Disease Research Laboratory (VDRL) test (Wiener Laboratorios, SAIC, Rosario, Argentina) and the Fluorescent Treponemal Antibody Absorption test (FTA-Abs, Inmunofluor Biocientifica SA, Argentina). Patients were considered to have syphilis when one of the tests (Dark field examination and/or serology) was positive.

### DNA extraction

Clinical samples (swabs, whole blood, and cerebrospinal fluids) were used for DNA isolation using a QIAamp DNA Minikit (Qiagen, Hilden, Germany) according to the manufacturer's recommendations.

### Detection of treponemal DNA

For the detection of treponemal DNA in clinical samples, a nested PCR amplification of the *polA* and *tmpC* genes was performed as described previously [15]. Samples were considered PCR-positive for treponemal DNA when PCR amplification was twice positive for at least one treponemal locus. To test whether the samples contained PCR inhibitors, the human gene encoding the enzyme methylenetetrahydrofolatereductase (MTHFR) was amplified using PCR in treponemal DNA-negative samples after the addition of human DNA as described previously [15].

### Molecular typing of treponemal DNA

SBMT was performed to type all positive samples as described previously [14]. Briefly, three loci including TP0136, TP0548, and 23S rRNA genes were amplified using the nested PCR protocol and the amplicons obtained were sequenced. Additionally, ECDCT was applied to all PCR-positive samples. This typing scheme included the determination of the number of 60-bp repetitions in the *arp* gene, Restriction Fragment Length Polymorphism (RFLP) analysis of the *tprE*, *G* and *J* genes, and sequencing of an 83-bp long fragment within the TP0548 gene (position 130–212 of TPANIC_0548; TPA Nichols; accession no. CP004010.2) as described previously [11, 12]. A DNA sample of the reference TPA Nichols strain served as the positive control for amplification of TPA DNA.

### DNA sequencing

PCR products of the TP0136, TP0548, and 23S rRNA genes were purified using a QIAquick PCR Purification Kit (Qiagen, Hilden, Germany) according to the manufacturer's instructions. The PCR products were sequenced on an automated capillary DNA sequencing system (GATC-Biotech AG, Constance, Germany). Sequence analyses were performed using Lasergene software (DNASTAR v. 7.1.0.; DNASTAR, Madison, WI). Sequences of 23S rRNA gene amplicons were evaluated only at positions 2058 and 2059 in the 23S rRNA gene of *Escherichia coli* (accession no. V00331), where A→G mutations are associated with macrolide resistance.

### Phylogenetic analyses

Phylogenetic trees were generated with MEGA 6 using the bootstrapping Maximum-likelihood algorithm, Tamura Nei model [16], and Network software with the Median Joining algorithm [17]. Both Maximum-likelihood and Median Joining algorithms were used for generation of trees from concatenated sequences of TP0136, TP0548, and 23S rRNA genes. Altogether, 1930 bp-long concatenated sequences corresponded to coordinates 303–1450, 110–868, 2100–2101 of TPANIC_0136, TPANIC_0548, and TPANIC_r0002 genes (numbering is based on the Nichols genome, CP004010.2). Concatenated sequences were analyzed for all samples that were typed using SBMT in the Czech Republic and in Argentina since 2006.

### Statistical methods

Analyses of statistical significance were performed using Fisher’s exact two-tailed test with STATISTICA software v.12 (StatSoft, Tulsa, OK). *P* values lower than 0.05 were considered to be statistically significant.

### Ethics statement

This study was approved by the Ethics Committee of the Hospital de Clínicas “José de San Martín,” Universidad de Buenos Aires and all patients provided their written informed consent. The study was conducted according to the principles expressed in the Declaration of Helsinki [18].

### GenBank accession numbers

Unique sequences identified in this study were deposited in GenBank under following accession numbers: KY550364- KY550367.

## Results

### Characteristics of patients and clinical samples

**Fig 1:**
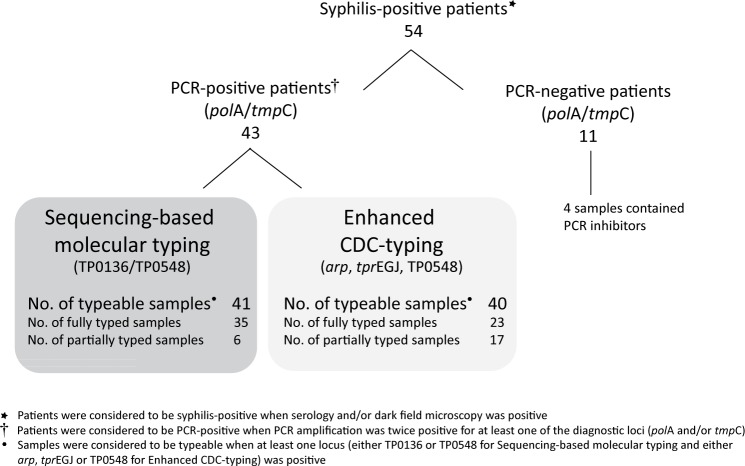
Flow chart of 54 patients included in the study.

A total of 54 samples (47 genital lesion swabs, four CSF, and three whole blood samples) were collected in 2006 and in 2013 from patients with syphilis. Out of 43 samples, where dark field microscopy was performed, 32.6% were positive. Except of two patients that were positive based on dark field examination with negative serology, all other patients were positive for serological tests (96.3%). Treponemal DNA (*pol*A and/or *tmp*C) was detected in 43 of the samples analyzed (79.6%) (Fig 1). External control was used to identify PCR inhibitors in all PCR-negative samples (see Methods section). PCR inhibitors were found in four samples (three CSF, one swab) and these samples were excluded from further analyses. Clinical characteristics of patients with syphilis diagnosis (based on serological and dark field microscopy examination) are presented in Table 1. Briefly, most of the patients were men with a mean age of 35 years, 56% of the individuals were MSM and approximately 46% had HIV infection. Regarding clinical status, most of the patients had primary syphilis at recruitment. No significant differences were observed between patients with positive PCR samples and those with negative PCR (Fisher’s exact two-tailed test) (Table 1).

**Table 1:** Characteristics of 50 patients enrolled in this study during 2006 and 2013 in Buenos Aires, Argentina.

Patient’s characteristics	All patients (n = 50)	PCR-positive patients (n = 43)	PCR-negative patients (n = 7)
Mean age in years (range)^a^	35.6 (17–66)	33.4 (17–67)	37.7 (19–66)
No. (%)			
Males	48 (96)	41 (95.4)	7 (100)
MSM^b^	27 (56.3)	23 (56)	4 (57.1)
HIV positive^c^	12 (46.2)	11 (47.8)	1 (33.3)
Primary syphilis	32 (64)	28 (65.1)	4 (57.1)
Secondary syphilis	10 (20)	10 (23.3)	
Congenital syphilis	1 (2)	1 (2.3)	
Undetermined stage	7 (14)	4 (9.3)	3 (42.9)
Genital, anal, oral swab	43 (86)	38 (88.4)	5 (71)
Condylomata lata swab	3 (6)	3 (7)	0
CSF	1 (2)	1 (2.3)	0
Whole blood	3 (6)	1 (2.3)	2 (29)

^a^age information of 5 patients was not available.

^b^MSM—Men who have sex with men; all investigated male patients declared their sexual orientation.

^c^HIV status was known only in 26 patients (52%).

### Sequencing-based molecular typing (SBMT)

Forty-three PCR-positive samples were analyzed using SBMT and 41 of them were typeable (95.3%). Samples were considered typeable when at least one sequence of the typing locus (either TP0136 or TP0548) was obtained. Samples from 35 patients were completely typed (including the TP0136, TP0548, and 23S rRNA genes sequences), while samples from six patients were only partially typed (Fig 1).

Altogether, 10 different TPA genotypes were found among the fully typed and partially typed samples; six were related to the sequence from the TPA strain SS14 (SSS, SU10S, SU11S, SSR9, SU2R8, SSR8), and four were related to the sequence from the TPA strain Nichols (U3U6S, U3U6R8, U3U12S, U5XS). All identified genotypes found in this study are shown in Table 2.

**Table 2:** Characteristics of detected genotypes using SBMT on 41 samples from syphilis PCR-positive patients from Buenos Aires, Argentina.

Genotype^a^	Typing	TP0136	TP0548	23S rRNA gene	Strain similarity	No. of isolates (%)
SSS	Complete	Identical to SS14	Identical to SS14	Sensitive	SS14-like	15 (36.6)
**U3U6S**^b^	Complete	Unique 3	Unique 6	Sensitive	Nichols-like	6 (14.6)
**SU10S**^b^	Complete	Identical to SS14	Unique 10	Sensitive	SS14-like	5 (12.2)
**SU11S**^b^	Complete	Identical to SS14	Unique 11	Sensitive	SS14-like	2 (4.9)
SSR9	Complete	Identical to SS14	Identical to SS14	A2059G	SS14-like	2 (4.9)
SU2R8	Complete	Identical to SS14	Unique 2	A2058G	SS14-like	2 (4.9)
U3U6R8	Complete	Unique 3	Unique 6	A2058G	Nichols-like	1 (2.4)
**U3U12S**^b^	Complete	Unique 3	Unique 12	Sensitive	Nichols-like	1 (2.4)
SSR8	Complete	Identical to SS14	Identical to SS14	A2058G	SS14-like	1 (2.4)
**XU6S**^b^	Partial	NA^c^	Unique 6	Sensitive	Nichols-like	1 (2.4)
**XU10S**^b^	Partial	NA	Unique 10	Sensitive	SS14-like	1 (2.4)
XSS	Partial	NA	Identical to SS14	Sensitive	SS14-like	1 (2.4)
U3XS	Partial	Unique 3	NA	Sensitive	Nichols-like	1 (2.4)
SXS	Partial	Identical to SS14	NA	Sensitive	SS14-like	1 (2.4)
**U5XS**^b^	Partial	Unique 5	NA	Sensitive	Nichols-like	1 (2.4)

^a^genotypes were denoted using SBMT by a tree-letter code [14]. Briefly, the first letter stands for the TP0136 sequence (S, identical to SS14; U, unique sequence compared to SS14 strain; X, not determined), the second letter stands for the TP0548 sequence (S, identical to SS14; U, unique sequence compared to SS14 strain; X, not determined), and the third letter stands for sensitivity/resistance to macrolide antibiotics (S, sensitive; R8, A2058G, resistant; R9, A2059G, resistant).

^b^new genotypes detected in this study (shown in bold).

^c^NA, not available.

**Fig 2:**
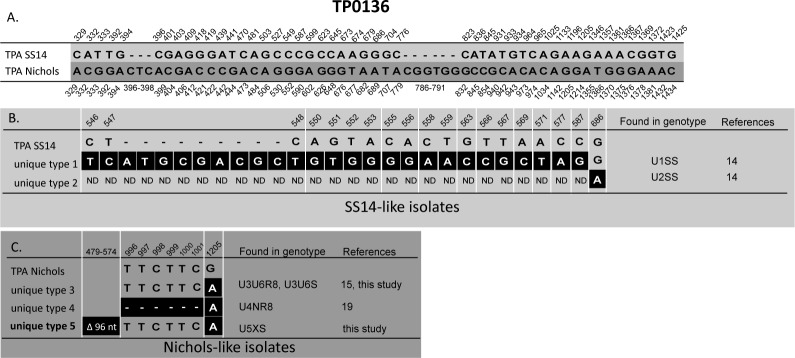
Alignment of TP0136 unique sequences identified to date [this study, 14, 15, 19]. Only positions containing nucleotide variants are shown. **A.** Nucleotide differences between reference strain TPA SS14 and TPA Nichols in the region characterized using SBMT (at coordinates 264–1469 according to the TPANIC_0136; CP004010.2). Coordinates shown above correspond to TPASS_0136 (TPA SS14; CP004011.1) and coordinates shown below correspond to TPANIC_0136 (TPA Nichols; CP004010.2). **B.** Nucleotide differences in SS14-like unique sequences. Coordinates correspond to TPASS_0136 (TPA SS14; CP004011.1). ND, was not determined due to the limited amount of sample DNA. The white lines between coordinates indicate the non-continuous nucleotide positions. **C.** Nucleotide differences in Nichols-like unique sequences. Coordinates correspond to TPANIC_0136 (TPA Nichols; CP004010.2). The new unique sequence identified in this study is shown in bold. The white lines between coordinates indicate the non-continuous nucleotide positions.

**Fig 3:**
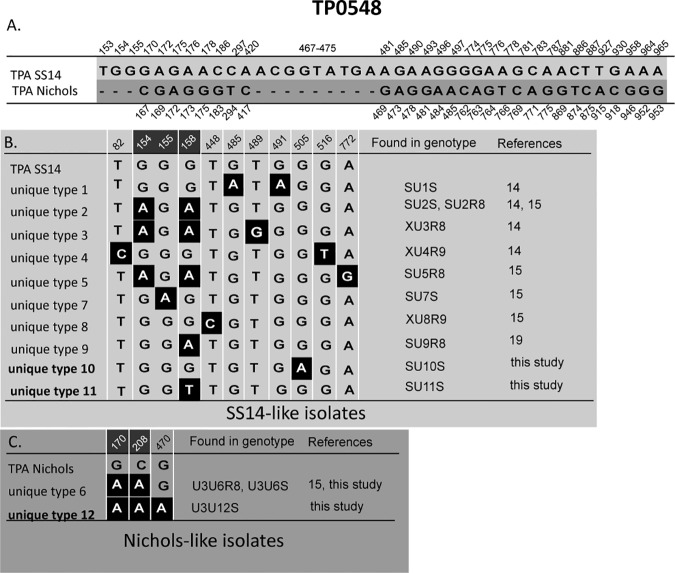
Alignment of all TP0548 unique sequences identified to date [this study, 14, 15, 19]. Only positions containing nucleotide variants are shown. **A.** Nucleotide differences between reference strain TPA SS14 and TPA Nichols in the region characterized using SBMT (at coordinates 16–1080 of TPANIC_0548; CP004010.2). Coordinates shown above correspond to TPASS_0548 (TPA SS14; CP004011.1) and coordinates shown below correspond to TPANIC_0548 (TPA Nichols; CP004010.2). **B.** Nucleotide differences in SS14-like unique sequences. Coordinates correspond to TPASS_0548 (TPA SS14; CP004011.1). New unique sequences identified in this study are shown in bold. The white lines between coordinates indicate the non-continuous nucleotide positions. Highlighted coordinates shown in white (154, 155, 158) correspond to regions determined by ECDCT. **C.** Nucleotide differences in Nichols-like unique sequences. Coordinates correspond to TPANIC_0548 (TPA Nichols; CP004010.2). New unique sequence identified in this study is shown in bold. The white lines between coordinates indicate the non-continuous nucleotide positions. Highlighted coordinates shown in white (170, 208) correspond to regions determined by ECDCT.

The most prevalent genotype (SSS) was found in 15 (36.6%) out of 41 typed isolates and was sequentially identical to TPA SS14. However, unlike strain SS14, isolates with the SSS genotype were sensitive to macrolides. Other prevalent genotypes included U3U6S (14.6%) which was related to the TPA Nichols, and the SU10S genotype (12.2%) which was related to the TPA SS14. In addition, genotypes SU2R8, SU11S, and SSR9 were detected in two independent clinical samples while other genotypes were detected in only one sample. According to either TP0136 and/or TP0548 analyses, treponemes in 30 clinical samples (73.2%) belonged to the SS14-like group and 11 (26.8%) belonged to the Nichols-like group. Moreover, five new genotypes (SU10S, SU11S, U3U12S, U3U6S, and U5XS) were detected and characterized in this study. Unique sequence U5 found at the TP0136 locus differed in a 96 bp-long deletion and one substitution compared to TPA Nichols. Unique sequences U10 and U11 found at the TP0548 locus each differed in one nucleotide position compared to TPA SS14. Unique sequence U12 found at the TP0548 locus had 3 substitutions compared to TPA Nichols. Alignment of TP0136 and TP0548 unique sequences are shown in Fig 2 and Fig 3.

### Enhanced CDC-typing (ECDCT)

ECDCT was applied to 43 PCR-positive samples and 40 were typeable: 23 were fully typed and 17 partially typed (Fig 1). Samples were considered typeable when at least one PCR amplicon (containing either *arp*, *tprEGJ*, or TP0548) was obtained. Altogether, 10 different genotypes were detected among the fully and partially typed samples (14d/d, 14d/f, 7d/f, 15d/f, 14d/g, 11d/f, 16d/e, Xb/f, 16p/X, Xk/f). Genotypes 14d/d and 14d/f were the most prevalent (15% each), followed by 7d/f (10%). Other genotypes, were detected in two (5% each) or in one sample (2.5% each) (Table 3.). Using this methodology, no new genotypes were detected.

**Table 3:** ECDCT genotypes detected in 40 samples from syphilis PCR-positive patients from Buenos Aires, Argentina.

Subtype^a^	Typing	No. of isolates (Frequency, %)
14 d/d	Complete	6 (15.0)
14 d/f	Complete	6 (15.0)
7 d/f	Complete	4 (10.0)
15 d/f	Complete	2 (5.0)
14 d/g	Complete	2 (5.0)
11 d/f	Complete	2 (5.0)
16 d/e	Complete	1 (2.5)
X d/f	Partial	6 (15.0)
X b/f	Partial	2 (5.0)
X d/d	Partial	2 (5.0)
16 p/X^b^	Partial	1 (2.5)
14 X/f	Partial	1 (2.5)
X d/X	Partial	1 (2.5)
X k/f	Partial	1 (2.5)
X X/d	Partial	1 (2.5)
X X/e	Partial	1 (2.5)
X X/f	Partial	1 (2.5)

^a^subtypes were denoted using ECDCT [11, 12]. Briefly, the number stands for number of repetitions in the *arp* gene, the first letter stands for type revealed from RFLP analysis of *tprE*, *G* and *J* genes and the last letter stands for the 83-bp long sequence of TP0548.

^b^X, undetermined.

### Prevalence of macrolide resistance-causing mutations in syphilis-causing treponemes

The 23S rRNA gene locus was amplified in 42 samples, and in six of them (14.3%), either the A2058G (four patients, 9.5%) or the A2059G (two patients, 4.8%) mutation was found. No strains harbored both A2058G and A2059G mutations. The A2058G mutation was present in samples isolated in 2006 and 2013, while the A2059G mutation was detected only in samples from 2013. Macrolide resistance was detected in both SS14-like (5/30) and Nichols-like (1/11) clinical isolates without significant differences (16.7% and 9.1%, respectively).

### Statistical analysis

We used the Fisher’s exact two-tailed test to examine associations between genotypes, TP0136/TP0548 alleles, Nichols-like/SS14-like groups, the *arp* locus or *tprEGJ* loci and macrolide sensitivity/resistance, MSM status, patient’s age, syphilis stage or time of isolation. No statistically significant associations were found (data not shown).

## Discussion

This study explored, for the first time, the characteristics of *Treponema pallidum* subsp. *pallidum* (TPA) circulating in Buenos Aires, Argentina. Besides the report on six TPA isolates from Colombia [20] and 14 TPA isolates from Peru [21], this is one of the first attempts to study TPA diversity in South America using a larger sample set.

**Fig 4:**
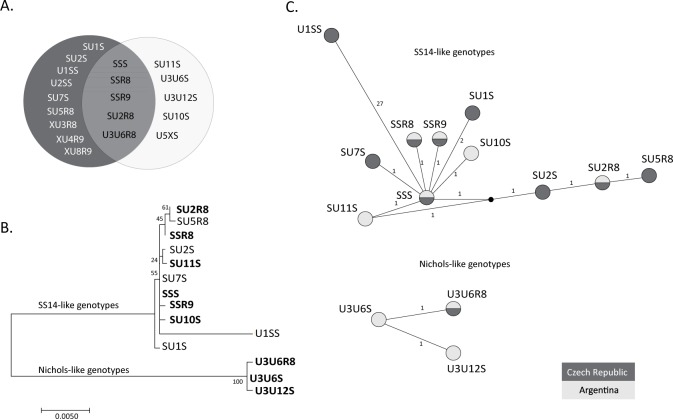
Genotypes identified using SBMT to date in the Czech Republic and in Argentina [this study, 14, 15] and their phylogenetic and network analyses. **A.** Genotypes identified exclusively in the Czech Republic (dark grey background), in Argentina (light grey background) and in both locations. Both fully and partially typed genotypes are shown. **B.** The phylogenetic tree of concatenated sequences of TP0136, TP0548, and 23S rRNA. Partially typed genotypes (XU8R9, XU3R8, XU4R9, and U5XS) were excluded. The U2SS genotype is also not shown due to the short sequence obtained from the TP0136 gene. Genotypes identified in this study are shown in bold. The bar scale represents 0.005 substitutions per target site. The numbers at each node correspond to bootstrap values. **C.** The network tree of concatenated sequences of TP0136, TP0548, and 23S rRNA genes. Partially typed genotypes (XU8R9, XU3R8, XU4R9 and U5XS) were excluded. The U2SS genotype is also not shown due to the short sequence obtained from the TP0136 gene. Every circle represents a different genotype. The small numbers represent the phylogenetic distances between genotypes. The black circle represents an unknown ancestor. Genotypes identified in the Czech Republic and in Argentina are shown in dark and light grey, respectively.

In our study, two different typing schemes were applied to TPA clinical isolates. Among samples from 54 patients with positive syphilis serology and/or dark field microscopy, the majority were characterized using ECDCT [11, 12] and SBMT [13, 14]. Altogether, 10 different treponemal genotypes were identified by both typing systems. SBMT revealed five new genotypes that were denoted using three-letter codes as described previously (SU10S, SU11S, U3U12S, U3U6S, and U5XS) [14] (Fig 4A). In contrast, no new subtypes were identified using ECDCT. This discrepancy likely reflects differences in the global number of clinical isolates characterized using the different methods; while altogether SBMT was carried out to type 175 samples [13–15, 19; this study], ECDCT was used to type almost two thousand clinical isolates [12, 21–32]. Another hypothesis is related to the fact that the typing efficiency was lower by ECDCT (53.5% of clinical isolates were fully typed) compared to SBMT (83.7%). Partial molecular typing obtained by ECDCT was mostly due to the low success rate of the *arp* gene PCR assay (failed in 15 out of 43 samples), something which has also been observed in other studies [33–35].

In this study, the most prevalent genotypes were 14d/d and 14d/f (15% each). Subtype 14d/f was also a predominant genotype in other countries including China [12, 23, 25, 27, 32], Peru [21], Russia [29], and the USA [12, 24]. On the other hand, subtype 14d/d was never observed to be a predominant genotype in any country where ECDCT was used [12, 21–32]. The genotypes previously found in Colombia and Peru [20, 21] were also identified in this study (except for the 13d and 22a, which were present in Colombia and 13X/f and XX/a genotypes, which were present in Peru). The most prevalent genotype detected using SBMT was SSS (36.6%) followed by U3U6S (14.6%), and the newly described SU9S (12.2%). This genotype distribution is different from that reported by Grillová *et al*. [15] in clinical samples from the Czech Republic, where SU2R8 was the most prevalent type (37.5%) followed by SSS (22.5%) and SU5R8 (15%). Several studies have shown that predominant treponemal strains in a particular population can change over time [12, 14, 15]. It has been described previously [14] that there is no relationship between genotypes identified using SBMT and ECDCT. In this study, treponemes with SBMT genotype SSS were further divided using ECDCT into subtypes 7d/f, 11d/f, and 14d/f. On the other hand, subtype 14d/f was further divided using SBMT into genotypes SSS, SSR8, and SU9S. All other SBMT genotypes were represented by a single ECDCT genotype and *vice versa* (Table 4.). These data suggest that the *arp* and *tprEGJ* loci are independent of TP0136 and TP0548. Moreover, higher variability at the *arp* and *tpr* loci have been found among different clinical isolates obtained from the same patient [36].

**Table 4:** Comparison of fully typed clinical isolates using ECDCT and SBMT. Only samples with fully-typed genotypes in both typing schemes are shown.

Genotypes/subtypes	7d/f	11d/f	14d/d	14d/f	16d/e	15d/f	14d/g
SSS	4	2		2			
U3U6S			6				
SSR8				1			
SU10S				3			
SU11S					1		
SSR9						2	
SU2R8							2

Based on whole and partial genome sequence analyses of laboratory TPA strains and clinical isolates, two genetically distinct groups were found [37–39]. Syphilis-causing strains were either genetically related to the reference strain SS14 (SS14-like strains) or to the Nichols reference strain (Nichols-like strains) (Fig 4B). Both, the 83bp region of TP0548 analyzed using ECDCT and the 1065 bp region of TP0548 analyzed by SBMT can be used to classify clinical isolates as either SS14-like or Nichols-like. So far, most of the sequentially characterized clinical isolates (n = 1746) belong to the SS14-like group (n = 1635; 93.6%) and only 6.4% belong to the Nichols-like group [12, 14, 15, 19, 21–32]. However, the geographic distribution of both groups differs. While the prevalence of clinical isolates containing Nichols-like treponemal DNA was 0% in the UK and Russia [22, 29] and very low (under 10%) in Australia, China, Czech Republic, Denmark, France, Ireland, and USA [12, 14, 15, 23–26, 30–32], a higher prevalence of Nichols-like treponemes was detected in Peru (21.4%) [21], Taiwan (20.2%) [28], and Madagascar, where, surprisingly, 100% of the typed clinical samples were Nichols-like [12]. In addition to Peru, Taiwan, and Madagascar, data from this study showed a high prevalence of Nichols-like isolates in Argentina (26.8%; based on TP0136 and/or TP0548). Since four different genotypes were found among 11 clinical isolates belonging to the Nichols-like TPA group in Argentina, the variability within Nichols-like clinical isolates appears to be similar to or greater than that found in the SS14-like group, where six different genotypes were found among 30 SS14-like clinical samples.

When the results obtained in this study were compared to previous SBMT studies performed in the Czech Republic [13–15], five distinct genotypes were present in both locations while nine genotypes were found only in the Czech Republic and five only in Argentina (Fig 4A and 4C). These results support the findings of other authors [12, 24, 30] i.e., unique subtypes exist in a particular geographic area and common subtypes exist globally. The MJ network analyses of genotypes from the Czech Republic and Argentina revealed that all the genotypes identified in the SS14-like group showed a star-like topology (Fig 4C). The central genotype is the most prevalent one (SSS), which is probably the origin of the rest of SS14-like genotypes found in clinical samples. The exceptions are SU2S, SU2R8 and SU5R8 genotypes, which arose probably independently from the SSS genotype from an unknown ancestor (Fig 4C). Interestingly, when the genotypes from a branch with a star-like topology were compared to genotypes with a linear topology, we found a statistically significant association (p = 0.039; Fisher’s exact two-tailed test) between SU2S, SU2R8, and SU5R8 genotypes and MSM patients, suggesting that these genotypes spread mainly among MSM patients.

In Argentina, we found macrolide resistance-associated mutations (either A2058G or A2059G) in only 14.3% of the samples analyzed. Although in clinical samples isolated from 2006 macrolide resistance-associated mutations were found only among Nichols-like genotypes; in samples obtained in 2013 macrolide resistance was found in both genetically distinct groups. There are several other geographical areas where the prevalence of macrolide resistance is still quite low and these areas include Canada (12.1%) [40–41], Russia (2.4%) [29], South Africa (1%) [42], Madagascar (0.7%) [24, 43], Taiwan (0.7%) [28], and Peru, where no macrolide-resistant mutations have been found among the 10 samples analyzed to date [21]. However, a high prevalence of macrolide resistance (61%–100%) has been reported around the world by different studies carried out in Australia, Cuba, Czech Republic, China, Ireland, UK, and the USA [14, 15, 22, 24, 32, 44–54]. Moreover, several studies have described an increasing trend in macrolide resistance in TPA clinical isolates [reviewed in 55]. In addition to the 10 genotypes, which were typed in at least two independent chromosomal TPA loci and showing identical typing results with different macrolide resistance genotypes (nine related to the SS14 strain and one to the Nichols strain) [30, 55], we also found that the U3U6 genotype (belonging to the Nichols-like group) had two variants in the 23S rRNA gene including a wild-type and A2058G genotype. This finding supports the scenario in which macrolide resistance emerged independently several times in both Nichols and SS14-like treponemes.

In conclusion, this is one of the first extensive reports on TPA molecular typing in South America. In it we analyzed clinical samples using two typing methods (ECDCT and SBMT). SBMT was used to detect five new genotypes as well as new genotypes within the Nichols-like group of TPA. Macrolide resistant TPA isolates were detected in South America at a relatively low frequency compared to studies from other geographical areas.
